# Greater Omentum Abscess Revealing an Upper Genital Infection

**DOI:** 10.3390/diagnostics15101261

**Published:** 2025-05-15

**Authors:** Romain L’Huillier, Alexandra Braillon

**Affiliations:** 1 Department of Medical Imaging, Edouard Herriot Hospital, Hospices Civils de Lyon, University of Lyon, 69002 Lyon, France; 2 LabTAU, INSERM U1032, 69003 Lyon, France; 3 Everest, The French Comprehensive Liver Center, Hospices Civils de Lyon, University of Lyon, 69002 Lyon, France; 4 Department of Medical Imaging, Louis Pradel Hospital, Hospices Civils de Lyon, 69002 Lyon, France

**Keywords:** epiploic abscess, pelvic inflammatory disease, greater omentum

## Abstract

In this clinical case, we report an upper genital infection revealed on Computed Tomography by a greater omentum abscess. The infection was confirmed by endocervical swabs and ultrasound-guided sampling of the epiploic abscess, which found the same bacteria (*Parvimonas micra*). Omental absecesses are most often secondary to spontaneous or post-operative infarction of the greater omentum, and this observation provides a new cause for epiploic abscesses.

**Figure 1:**
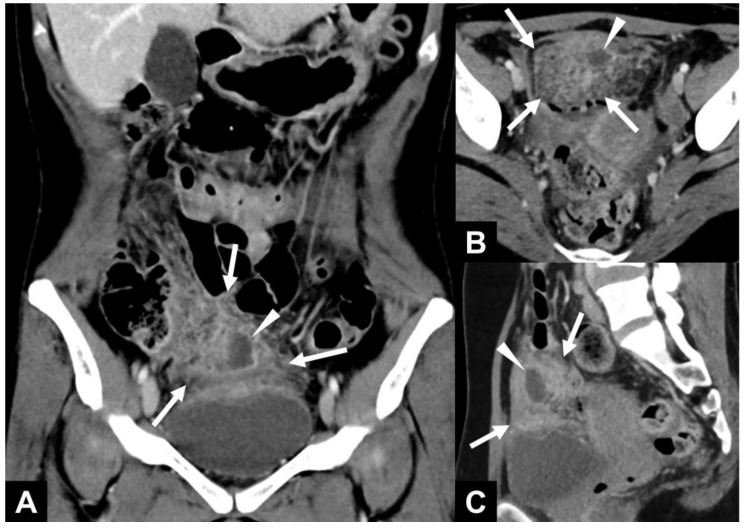
A 41-year-old female patient with no surgical history presented with pelvic pain after the removal of an intrauterine device 4 days ago, without fever and with an increased CRP level (188 mg/L). Clinical examination revealed pain provoked by a vaginal exam, and endovaginal ultrasonography revealed only a small amount of pelvic effusion while the ovaries and fallopian tubes were considered normal. Because of the significant inflammatory syndrome, it was decided to perform an abomino-pelvic CT. Conventional CT (SOMATOM^®^ Definition Edge, Siemens Healthineers, Erlangen, Germany) was performed after the intravenous injection of an iodinated contrast medium in the portal phase. Abdomino-pelvic CT at the portal phase in coronal (**A**), axial (**B**), and sagittal (**C**) reconstructions revealed enlargement and stranding of the lower part of the greater omentum (white arrows) associated with a developing collection within it (white arrowheads). The upper wall of the bladder showed reactive thickening.

**Figure 2:**
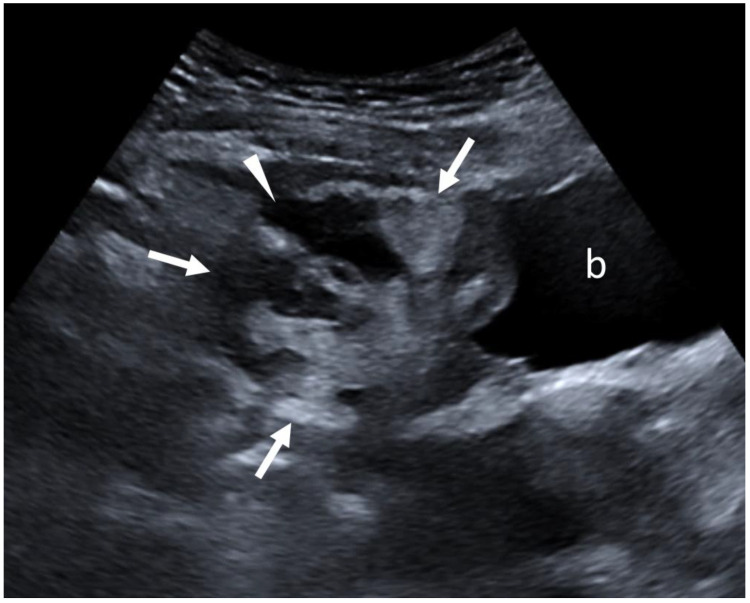
On ultrasound, in a sagittal orientation, the distal part of the greater omentum (white arrows), just above the bladder (b), appeared heterogeneous and was hyperechoic with an anechoic liquid zone corresponding to the abscess in formation (white arrowhead). An ultrasound-guided puncture of the epiploic fluid collection was performed the following day, which confirmed the diagnosis of epiploic abscess with the same bacteria as identified in the endocervical samples. The final diagnosis was therefore that of an epiploic abscess secondary to an upper genital infection with *Parvimonas micra*. The patient received triple antibiotic therapy (ceftriaxone, doxycycline, and metronidazole) for 15 days and was discharged after 3 days in the hospital and went home. Biological inflammatory syndrome disappeared in three days, and a follow-up ultrasound at one month showed complete regression of the greater omentum fluid collection. Omental abscesses can be primary [1] or, most commonly, secondary to spontaneous or post-operative infarction [2,3]. Omental infarction is usually due to venous insufficiency secondary to trauma, abdominal surgery, or venous thrombosis of the veins of the greater omentum [4,5]. Reactive involvement of the greater omentum has already been described in the context of severe pelvic inflammatory disease with tuboovarian abscess, which acts as an internal barrier to contain the infectious process in the pelvis in case of peritonitis [6]. Although MRI is the most accurate imaging modality for the diagnosis of pelvic abscesses, particularly those secondary to pelvic inflammatory diseases [7], CT and ultrasound are easily accessible modalities that allow for the suspicion of the diagnosis in this clinical case. This observation shows the possible occurrence of a greater omentum abscess secondary to a non-severe pelvic inflammatory disease.

## Data Availability

The data presented in this study are available upon request from the corresponding author. The data are not publicly available as they contain confidential doctor and patient information.

## Abbreviation

The following abbreviation is used in this manuscript:

CT	Computed Tomography
